# Rapid Spread of African Swine Fever Across Borneo

**DOI:** 10.3390/ani15172529

**Published:** 2025-08-28

**Authors:** Olivia Z. Daniel, Sui Peng Heon, Christl A. Donnelly, Henry Bernard, C. David L. Orme, Robert M. Ewers

**Affiliations:** 1 Georgina Mace Centre for the Living Planet, Department of Life Sciences, Imperial College London, Silwood Park, Ascot SL5 7PY, UK; 2 South East Asia Rainforest Research Partnership, Danum Valley Field Centre, Lahad Datu 91112, Malaysia; 3 School of Public Health, Imperial College London, London W12 0BZ, UK; 4 Department of Statistics, University of Oxford, Oxford OX1 3LB, UK; 5 Pandemic Sciences Institute, University of Oxford, Oxford OX3 7BN, UK; 6 Institute for Tropical Biology and Conservation, Universiti Malaysia Sabah, Jalan UMS, Kota Kinabalu 88400, Malaysia

**Keywords:** African Swine Fever, bearded pig, Borneo

## Abstract

African Swine Fever (ASF) reached the island of Borneo at the end of 2020. The first mortalities were recorded in wild bearded pigs (*Sus barbatus*) in Sabah, north-east Borneo. The virus then began to spread across the island but due to COVID-19 lockdowns the spread was difficult to monitor on the ground. With the urgent need to track this epidemic, and in the absence of traditional monitoring, the Babi Hutan Project was launched in April 2021 to gather data on pig sightings using citizen science. Any sightings of bearded pigs were requested via the website, social media and a WhatsApp hotline. Here we bring together the data from this project and other online sources to show how the virus spread across almost the entire island within a one-year period. The speed of spread appeared to increase with time following an exponential model: we estimate an average speed of spread of 0.89 km/day after 100 days since the first observation and at 4.28 km/day after 400 days. Our key recommendations are: that existing hunting bans on bearded pigs remain in place; that urgent biosecurity measures should be put in place if outbreaks occur in areas with backyard (domestic) pigs; that surviving pigs are tested for resistance; that the disease dynamics are modelled and that the International Union for Conservation of Nature (IUCN) urgently re-evaluates the bearded pig’s status.

## Simple Summary

Bearded pigs are important both ecologically and economically on the island of Borneo: they act as ecosystem engineers, and they are an important food source for many communities. At the end of 2020, people began to notice large numbers of bearded pigs were dying; shortly thereafter, domestic pigs began to die, and in early 2021 it was confirmed that African Swine Fever had arrived on the island of Borneo. Then lockdowns came into force due to COVID-19, making on-the-ground monitoring impossible. We therefore set up the Babi Hutan Project to centralise the reporting of pig mortalities through citizen science (conducting research with the participation of the general public). Integrating citizen science and online reports, the mortalities of more than 370 bearded pigs and more than 140,000 domestic pigs were recorded between 2020 and 2023. From these data, we have been able to track the spread of the virus across the island of Borneo. However, this virus did not eliminate bearded pigs. We also include recommendations for the next steps for the bearded pigs of Borneo.

## 1. Introduction

African Swine Fever (ASF) is a highly contagious viral haemorrhagic disease affecting members of the family Suidae (pigs, hogs and boars). In its acute form it can cause 95–100% mortality [1,2,3]. There is currently no approved vaccination and no cure [3,4]. It is a notifiable disease for the World Organisation for Animal Health (OIE/WOAH), and documentation of its spread is regularly updated on the Food and Agriculture Organisation of the United Nations (FAO) website [2,5,6]. Fortunately, it is not zoonotic.

The ASF virus is highly contagious: it can be transmitted directly through contact with infected pigs, by contact with bodily fluids of infected pigs, by ingestion of infected meat, and there is evidence that it can be airborne over short distances [3]. ASF can persist in frozen pig meat for over 100 days and dry-cured pork for over 80 days [7]. It can also be transmitted via a vector—a soft-bodied tick in the genus *Ornithodoros* [8]—however, this genus is not present in Southeast Asia [9]. Indirect transmission via fomites (inanimate objects that can be contaminated with pathogens and transmit them to a new host, such as wheels of vehicles, equipment, clothing, etc.) contaminated by the bodily fluids of infected pigs is considered possible, although further investigation is required for this to be substantiated [3,7].

African Swine Fever was endemic to Sub-Saharan Africa until 1957 [10], since then there have been several outbreaks around the globe, but these were mostly eradicated by 1979 [3]. Then, in 2007, ASF was introduced into Georgia, from where it spread across Europe, arriving in Asia in 2018 [5]. ASF was confirmed on the island of Borneo in early February 2021 [2,6,11]. The first cases of ASF were seen in bearded pigs (*Sus barbatus*) in the Malaysian state of Sabah, in the north of Borneo [6].

Prior to the ASF outbreak in Borneo, bearded pigs were listed as Vulnerable by the International Union for Conservation of Nature’s (IUCN) Red List of endangered species [12], and are recognised as ecologically and economically vital [13]. Bearded pigs are cited as ‘ecosystem engineers’, dispersing and predating tree seeds, rooting through soil and removing saplings [14,15]. They are also prey for apex predators such as the Sunda Clouded Leopard (*Neofelis diardi*) [16,17]. On Borneo, traditional hunting of bearded pigs dates back over 35,000 years [18], and before this ASF outbreak their meat represented 50 to 75% of hunted animal biomass, comprising an important protein source for many non-Muslim communities [13,19,20].

In Sabah, an unusually high number of dead bearded pigs were observed from December 2020 in Kinabatangan [21]. ASF was confirmed to be the cause of pig mortalities by the OIE in February 2021 in Pitas [6,11], the bodies of pigs tested positive for ASF by the Veterinary Research Institute using real-time polymerase chain reaction (RT-PCR) tests [6]. Historically, bearded pig populations on Borneo have not been directly monitored, further complicating collating data on the impact of ASF. Limited access to field sites as a result of COVID-19 restrictions also precluded formal rapid response surveillance.

African Swine Fever can spread to any pigs, however, including domestic pigs. In 2020 there were 45 commercial pig farms registered in Sabah [22], as well as 282 ‘backyard pig’ farms [23]. The latter refers to village pigs or Babi Kampung, which are raised in small holdings using subsistence or traditional methods, where they will often be allowed to roam around the villages and are usually slaughtered and consumed within the villages [22]. Reports of dead domestic pigs followed those of bearded pigs in Sabah and beyond into Sarawak and into Indonesian Borneo [2]; within 12 months of ASF officially being recorded on Borneo the virus had encompassed almost the entire island.

## 2. Materials and Methods

In April 2021, the Babi Hutan Project was launched, a collaboration between Sabah Wildlife Department, Sabah Veterinary Service and Imperial College London, to track the spread of ASF through wild bearded pig populations using citizen science [21]. Sightings of bearded pigs, either dead or alive, were requested via the website, social media, and a WhatsApp hotline. The website had a link to a questionnaire which asked for details of the location, the date, the species, the number sighted (0–5, 5–10, 10–20, 20–30 and >30) and if they were alive or dead (see Figure S1 in the Supplementary Materials for a copy of the questionnaire). The project also sought online sources of data on reported pig mortalities in Borneo, noting if they were bearded pig or domestic pigs. For the questionnaires and reports received directly by the Babi Hutan Project, when quantifying the reports of pigs seen, we used the lowest number in the range (so if the range was 5–10, we counted this as 5), and when people reported having seen ‘many’ dead pigs we counted this as 5 pigs. All reports of dead pigs received by the Babi Hutan Project were passed on to the relevant wildlife and veterinary departments. At the time of publication, the OIE has only published data on ASF cases in Sabah and Sarawak, and the majority of these document domestic pigs: of the 76 outbreaks listed, 70 were sampled and all tested positive for ASF using RT-PCR testing [6,24]. Reports of pig deaths in Indonesian Borneo are listed on the FAO website; these are mainly via links to documents and websites that report outbreaks, but again refer almost entirely to domestic pigs [2]. In addition, iSIKHNAS (Indonesia’s National Animal Health and Production Information System) is an animal health information system owned and managed by the Indonesian Ministry of Agriculture, which has reports of ASF cases, giving locations and number of mortalities, although it does not specify whether they are bearded or domestic pigs [25]. The testing methods used to confirm the cause of death as ASF were not specified on the iSIKHNAS website. Data were also gathered from local newspapers (86 reports) and local government reports (14 reports) documenting ASF spread to new areas. These data, along with OIE (75 reports), iSIKHNAS reports (276 reports) and the reports directly to the Babi Hutan Project (210 reports), have been compiled into a single database which includes both bearded and domestic pig deaths [26]. Of the 210 reports to the Babi Hutan Project, 144 were from local public agencies, 14 from oil palm plantation workers, 28 from research or conservation organisations and 24 from members of the public.

Compiling the data on all locations in Borneo and of both wild and domestic pigs, we have been able to quantify the rate of spread of the ASF virus across the island. This was achieved by plotting the date of the first reported death by district and distance from the first observation: the date of the first reported death in each district is either the first observation or the earliest reported date at a district level, using mid-month if only the month was recorded.

## 3. Results

**Figure 1:**
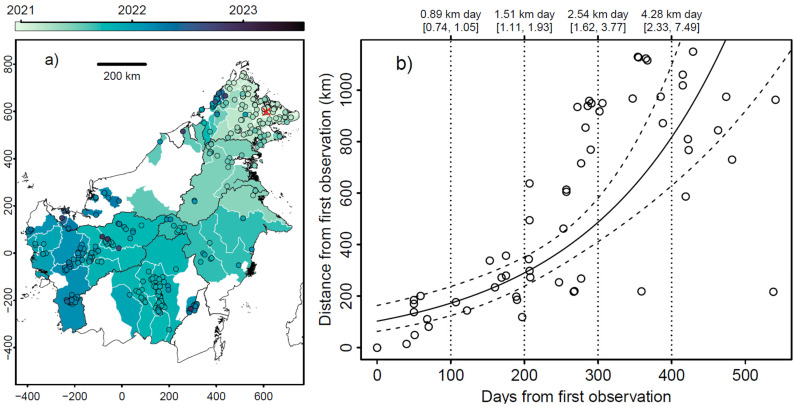
(**a**) Map showing the spread of African Swine Fever (ASF) across Borneo, coloured by the date of first occurrence recorded in each district. All reports of pig deaths due to ASF that had exact locations are superimposed as circles, and the first occurrence recorded by the Babi Hutan Project is indicated by a red cross. The lightest shading is for the earliest reports in December 2020, getting darker as time goes by, with the last report in July 2023. This map includes data from both domestic and bearded pigs. Map units are km in the UTM50N projection. A map and table with the names of the different regions and districts of Borneo can be found in Supplementary Materials Figure S2 and Table S1. (**b**) Scatterplot of date of first reported death, by district and distance from the first observation (the explanatory variable, as indicated by the red cross in Figure 1a). The date of the first reported death in each district is either the first observation or the earliest reported date at a district level, using mid-month if only the month was recorded. The solid fitted line shows the linear model and 95% CI (dashed lines). The vertical dashed lines are used to indicate the speed of spread (slope of the fitted model) and 95% confidence intervals for four time points.

To date, we have collated just over 660 reports of pig deaths across Borneo, amounting to 373 individual bearded pigs and more than 140,000 domestic pigs. Our data indicate that ASF swept rapidly through Borneo, expanding from north-east Sabah to the southern districts in Central Kalimantan, and districts on the south-west coast of West Kalimantan, in a period of just 12 months from when the virus was officially recognised (Figure 1a), although it is possible that the spread was quicker. Although we cannot discount the possibility that this rapid spread was aided by multiple incursions of ASF, Khoo et al. (2021) demonstrated that the ASF virus found in three locations in Sabah were identical to each other and of the same strain (p72 genotype II ASFV strain), which matches that in the Asia–Pacific region and indicates a common origin [27].

### 3.1. Sabah, Malaysian Borneo

**Figure 2:**
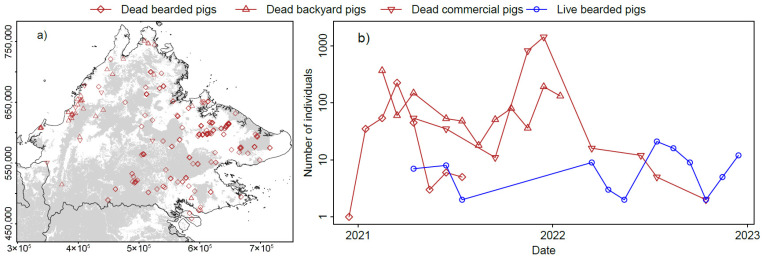
(**a**) A zoomed-in map of Sabah with locations of bearded pig (diamonds), backyard pig (triangles pointing up) and commercial pig (triangles pointing down) mortality between December 2020 and October 2022; forest cover is shown in grey [28]. Administrative boundaries are from GADM 4.1. (**b**) Reports of numbers of dead backyard pigs, dead commercial pigs and live and dead bearded pigs in Sabah, Malaysian Borneo, between December 2020 and December 2023. Data collated by the Babi Hutan Project [21].

In Sabah, the first mortalities were seen in bearded pigs in December 2020 and continued until July 2021; reports of backyard pig mortalities were recorded from February 2021 until January 2022; and commercial pig farms confirmed cases from April 2021 with outbreaks reported until October 2022 (Figure 2a,b). Between February 2021 and October 2022, 73 outbreaks documenting 4045 cases of ASF were reported to the OIE in Sabah [6], with no further cases reported until 2025 [24]. Bearded pig mortalities were reported from north, east, central and south Sabah, commercial pig mortalities were strongly concentrated in the west and backyard pig mortalities were spread across Sabah (Figure 2a). The citizen science data on observed pig mortality and live pig sightings provides quite coarse data. Some respondents used broad terms like ‘many’ and the largest numerical category does not have an upper bound (‘more than 30’). The minimum count is easiest to define, although it does provide more conservative estimates. Use of broad terms or numerical ranges affected just over 5% of the total collated reports of bearded pig deaths and 18% of reports of live pigs.

### 3.2. Sarawak, Malaysian Borneo

In Sarawak, cases were reported in areas bordering Sabah and North Kalimantan in July 2021 with a second wave of cases between January and October 2022 (Figure 1a). No further cases have been listed by the OIE since 2022 [6]; however, there have been isolated reports of outbreaks as recently as 2024 [29,30].

### 3.3. Kalimantan, Indonesian Borneo

The first reports of pig mortalities in Kalimantan (Indonesian Borneo) were in May 2021 [25,31] (Figure 1a). These were in both North and East Kalimantan and in September 2021 in both Central and West Kalimantan (Figure 1a). The most recent outbreak was in 2024 [25]. At the time of publication, no cases have been reported in South Kalimantan.

### 3.4. Brunei, Borneo

No cases have officially been reported from Brunei (Figure 1a).

### 3.5. Rate of Spread

Assuming that all ASF virus in Borneo since 2020 originated from the first observation in the north-east of Sabah, it is possible to model the rate of spread from that date and that location (Figure 1b). We used a linear model to fit log distance from first observation (d) as a function of the time since the first observation (t), generating an exponential model of the rate of spread. The model has good explanatory power (adjusted R^2^ = 0.54) and the slope and intercept are both highly significant (*p* < 0.0001). The modelled equation parameters and standard errors are ‘d = exp(4.63 [±0.186] + 0.00519 [±0.000631] t)’. The slope of this equation gives the speed of spread at a given time, and we refitted the model to bootstrapped data (n = 100,000) to estimate the 95% confidence intervals around both the fitted model and the estimated speed of spread for four time points during the epidemic. This model indicates that 100 days after the first observed mortality the rate of spread was 0.89 km per day, 1.51 km per day at 200 days, 2.54 km per day at 300 days and slowed to a rate of 4.28 km per day at 400 days.

## 4. Discussion

Malaysia has enacted import bans on live pigs and pig products from countries known to have ASF outbreaks from 2018 [32]. Although it is likely that the ASF virus first arrived in Borneo through infected pork products, in swill feed or the importation of infected domestic pigs [27], it is unlikely that the origin was mainland Malaysia as the first case of ASF was not reported there until October 2021 [33]. It is possible that certain traits of the bearded pig may contribute to the epidemiology of ASF, such as their living in family groups, their migratory tendencies and their consumption of carrion [34]. However, the incubation period of ASF in bearded pigs is unknown so their role in facilitating the spread of ASF cannot be characterised [34]. Ticks of the genus *Ornithodoros* are not found in Southeast Asia [9], so are not a possible vector.

To date, there are no formal estimates quantifying the magnitude of the population crash (a sudden sharp reduction in the size of a population) that bearded pigs have suffered due to ASF. Officially reported cases only represent ASF cases verified by positive laboratory test results. It has been estimated that 90% of Sabah’s bearded pig population disappeared [35], and there are anecdotal reports that the situation may be similar in Sarawak’s bearded pig populations [36], which is in line with ASF outbreaks in other wild pig populations [37]. Reports of pig footprints and wallows started to come in, as well as sightings of low numbers of live bearded pigs in Sabah, mid-way through 2022 (Figure 2b); these included sightings of juveniles, indicating that survivors had reproduced.

In the absence of COVID-19, proper surveillance could have been set up using camera traps and dung transects to try to establish the levels of bearded pig decline. However, as these methods were not possible due to lockdowns it was decided that citizen science was the only method for gathering information on mortalities. It is possible that using this method created bias in that only people aware of the project would make reports. However, maximum effort was made to publicise the project with a dedicated website and Facebook group, as well as tapping into both government and conservation project networks. In addition, palm oil plantations were contacted. Outreach from the project was conducted in English, Malay and Indonesian. Indeed, it is possible that a broader range of reports were collected using this method as it would not have been possible to set up on-the-ground monitoring over such a large geographical area. Also, undertaking on-the-ground experiments and surveillance requires permits and permissions which can take up to six months to be approved, which may have resulted in missing the most dramatic decline of the bearded pigs in Sabah. As bearded pigs are the only wild pig species in Borneo, there was no risk of misidentification.

It is possible that in opening up reporting to members of the public using citizen science, some pigs that had died of other causes may have been reported as ASF cases to the Babi Hutan Project. To gather as much information as possible to allow us to determine if the reported mortalities were caused by ASF, the questionnaire had some open-ended questions such as if the pigs looked sick; photos were also encouraged (see Supplementary Materials). Some reports included photos where red patches, indicating internal haemorrhaging, could be clearly seen; this can be a symptom of ASF [38]. All reports of pig mortalities received by the Babi Hutan Project were passed on to the relevant wildlife and veterinary authorities; however, we did not receive test results for all reports received. In the absence of official test results, it cannot be verified that all the deaths reported were caused by ASF; however, there have been no other epidemics of known pig virus or disease (such as Classical Swine Fever, Porcine Reproductive and Respiratory Syndrome, Swine Influenza virus or Nipah virus) outbreaks reported in Borneo in recent years [39,40].

Hunting licences for bearded pigs have been suspended in Sabah since February 2021 [41]. It is essential that the current ban is enforced and that educational materials are made available to ensure that those that usually hunt bearded pigs are aware of the existence and purpose of the ban. It is also essential to ban the sale of bearded pig meat and to educate the general public to avoid consumption; this would reinforce the continuation of the suspension of hunting licences and would increase public awareness.

It is important to continue the implementation of existing ASF prevention protocols [38]—including controlled movement of pigs and pork products between states and countries, awareness campaigns, and close monitoring activities in farms and abattoirs. However, lessons should be learnt from the ASF outbreak in Borneo and also Peninsular Malaysia, where inadequate biosecurity practices were identified as key risk factors in the spread of ASF between domestic pig farms [42]. A key recommendation was to move from open pig houses to enclosed houses on pig farms [42]. Transmission between bearded pigs and smallholders’ ‘backyard’ domestic pigs is possible due to poor biosecurity [34]. With this in mind, particular care should be taken by backyard pig owners when there are outbreaks, including village-level biosecurity practices and penning in pigs [34]. Until the epidemiology of ASF in bearded pigs is fully understood, it may be prudent to review the ASF prevention protocols to ensure adequate advice is available and tailored to the different farming practices to reduce the risk of allowing ASF to spill over from domestic settings to wild pigs, especially if it is possible that they could act as reservoirs between free-ranging small holdings [34]. For diseases such as ASF which have no cure, prevention of spread is key; therefore, any pig death or even pig illness should be investigated immediately [3]. Moreover, necessary precautions should be taken to ensure that ASF does not reach the bearded pig populations on the smaller islands that surround Borneo and indeed other islands with endemic pig populations, as Southeast Asia represents an important area for pig diversity with 11 endemic species found in the region [13,43].

Development of a vaccine for domestic pigs is closer than for wild pigs. There are many institutions globally working on developing an efficient vaccine for domestic pigs; however, due to the complexity of the virus, to date none have proved viable [44]. Indeed, the WOAH highlights the importance of high-quality and safe vaccines being trialled in the field due to the risks of inadvertently creating new ASF virus strains; they have therefore created an ASF vaccine standard [45]. A developed vaccine for domestic pigs would then need to be adapted and trialled on wild pigs; most vaccines under development need to be administered by injection [46], which would be impractical for wild populations. It is likely that oral vaccinations would be required for wild pigs in the form of bait, but this would come with additional complexities such as correct dosage and how to keep the vaccine stable in varying environmental conditions [46].

It is likely that the loss of such a large proportion of this keystone species will have far-reaching consequences, some of which became apparent shortly following their local disappearance and others that may not be realised for many years. It is possible that some of the more immediate ramifications resulting from the mass loss of bearded pigs in Borneo include the increased incidence of crocodile attacks on humans [47], as well as the decrease in diversity of dung beetles [48]. The effects on the tree populations may not become apparent for several years, but it is likely that without the seed predation by bearded pigs there will be a loss in diversity of tree species, as has been observed in areas in Borneo where they have become extinct [49].

## 5. Conclusions

We suggest a research agenda that can help guide further decision-making regarding control of the ASF outbreak. The continued collection and collation of bearded pig sightings and monitoring data are urgently required to obtain up-to-date information on the abundance and age structure of the remnant population. Such data are needed across the breadth of Bornean land uses, including protected areas, managed agricultural land, and forest remnants in the vicinity of domestic pig farms. We urgently need confirmation, via blood samples, as to whether bearded pigs have survived because they have been resistant or because they have evaded infection by the virus responsible for ASF. To do this, baited live traps could be set up where known populations have been captured on camera traps and blood samples could be extracted from healthy individuals. A serological assay, ELISA (Enzyme-linked Immunosorbent Assay), could be run on the blood to screen for ASF antibodies, which would indicate previous exposure to the virus. Also, if a freshly dead pig is reported to the authorities, a PCR test could be run on the blood from the fresh carcass to screen for the presence of viral DNA. It would also be prudent to collect any ectoparasites from captured pigs to determine if they contained any trace of the ASF virus and could act as potential vectors. ASF disease transmission models [50] should be developed and adapted to the Bornean landscape to help identify potential refugia and to guide further decision-making processes. Finally, the IUCN Red List status of the bearded pig should be re-evaluated urgently [13]. Given the suspected 90% decline in population, we would recommend reclassification from Vulnerable to Critically Endangered based on criterion CR.A.1.e. (suspected population decline of over 90%, which is clearly reversible and understood and ceased, based on pathogens [51]). The sharp decrease in their population makes them vulnerable to stochastic events, illegal hunting or another outbreak of AFS or other pathogens. However, bearded pigs can be very prolific breeders, with average litters of 7–9 piglets and females becoming receptive to mating within a week of giving birth; in mast years they have been known to raise 2–3 litters [52].

We are cautiously optimistic that ASF has not spelt the end for the bearded pigs of Borneo. We hope that if the survivors are given the chance to repopulate, these engineers of Borneo’s forests can resume their work.

## Data Availability

The database used for this work has been published on Zenodo (https://zenodo.org/records/10568985) (accessed on 21 May 2025). It is under embargo until January 2026.

## Supplementary Materials

The following supporting information can be downloaded at: https://www.mdpi.com/article/10.3390/ani15172529/s1, Figure S1: Copy of the Babi Hutan Project questionnaire, Figure S2: Map of country, province and district names in Borneo, Table S1: District name gazetteer for Borneo.

## Abbreviations

The following abbreviations are used in this manuscript:

AFS	African Swine Fever
OIE/WOAH	World Organisation for Animal Health
FAO	Food and Agriculture Organisation of the United Nations
IUCN	International Union for Conservation of Nature
