# Supplementary material for: Rapid Spread of African Swine Fever Across Borneo
Source: Animals (Basel). 2025 Aug 28;15(17):2529. doi: 10.3390/ani15172529 (PMC12427262; doi:10.3390/ani15172529)
Supplement: Supplementary file 1 [file animals-15-02529-s001.zip › animals-3790056-supplementary.pdf]

Despite not posing a risk to humans, African Swine Fever is currently a major threat to the survival of the bearded pig. To stop the spread of African Swine Fever in Malaysian Borneo, the Babi Hutan Project want your sightings of both bearded and domestic pigs – both dead or alive – to understand how far and fast it is spreading. Please help the Babi Hutan Project by reporting any pig sightings using this form. For further information please go to our website at https://www.babihutan.com/

Where was the pig? (Name of area/estate/village/park)

Can you provide a postcode for the sighting? (Optional)

When did you see the pig? (DD/MM/YY)

What type of pig did you see? (see image below for reference of Bearded Pig)

- ○ Domestic pig
- ○ Bearded Pig
- ○ Village Pig

Left: Bearded Pig, Right: Domestic Pig

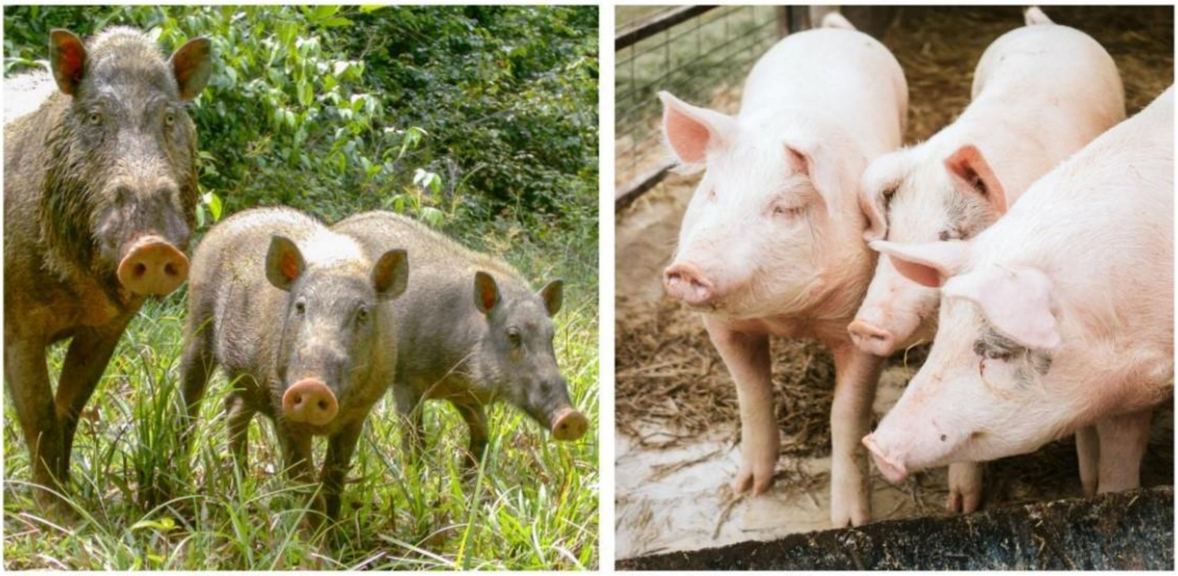

Photo: Oliver Wearn / SAFE Project

Photo: Amber Kipp / Unsplash

How many pigs did you see?

- ○ 0
- ○ 1-5
- ○ 6-10
- ○ 10-20
- ○ 20-30
- ○ 30+

Were the pigs alive or dead?

- Alive
- Dead
- Some alive and some dead

If the pigs were alive, did they look sick? (Optional)

Are you able to provide any photos? Please do not risk touching any pigs to get photos. (Optional)

These data are being collected as a joint research initiative between the Sabah Wildlife Department, Sabah Veterinary Service, and Imperial College London

Your sighting has been recorded. If you have come into contact with an infected pig, please thoroughly disinfect yourself and any vehicles you have travelled in to help further stop the spread of African Swine Fever. Thank you from all of us at the Babi Hutan Project for your help!

Figure S1. Babi Hutan Project Questionnaire.

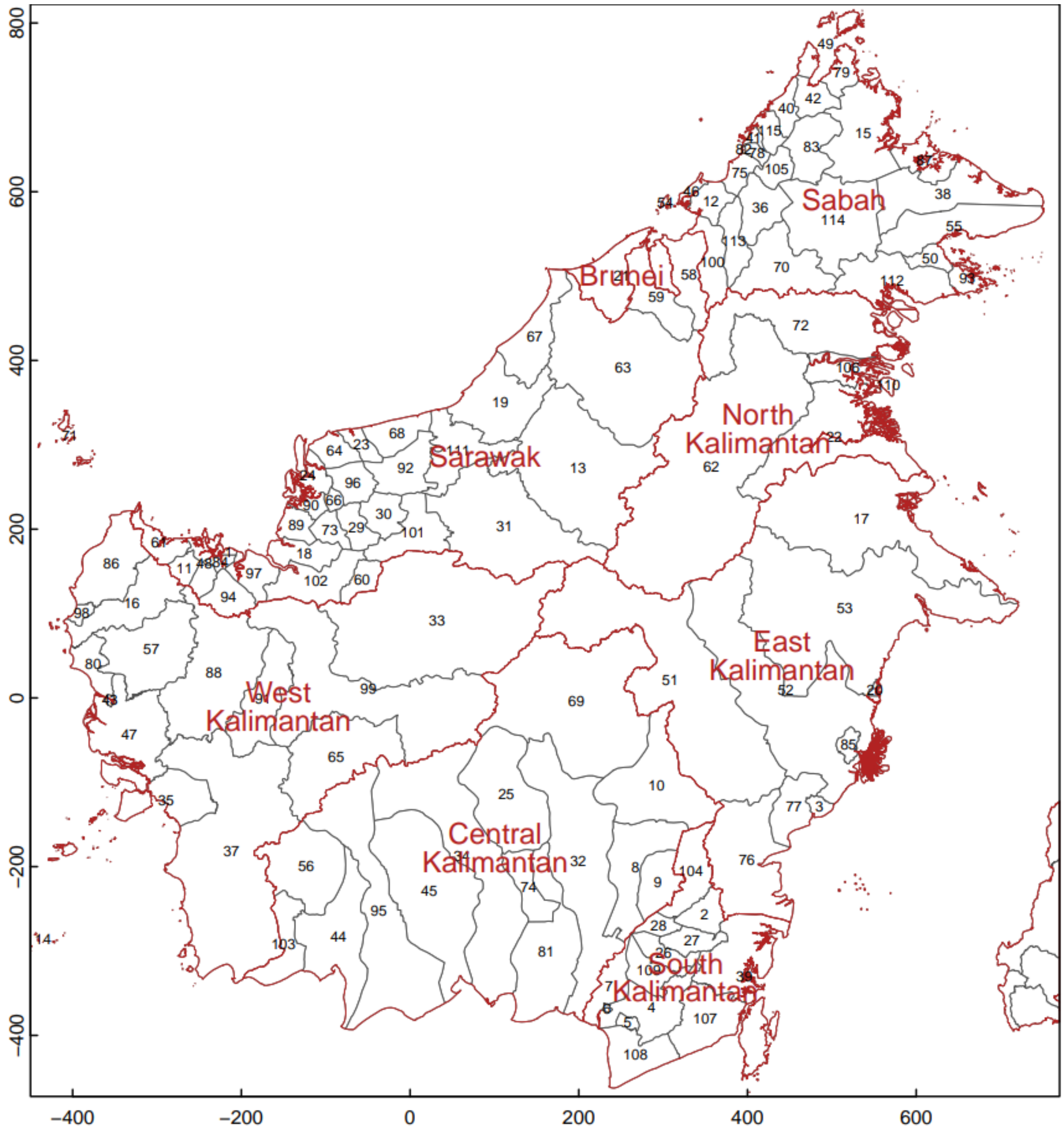


Figure S2. Map of country, province and district names in Borneo. The red labels show the location of Brunei and the provinces of Malaysian and Indonesian Borneo. The black numbers label the individual districts used in mortality reporting and Table S1 provides matching district names. The map units are kilometres and the map uses the UTM50N projection.

Table S1. District name gazetteer for Borneo. See Figure S1 for the locations of each district. The code numbers are ordered by alphabetic district name.

| Code | Country | Province | District |
|---|---|---|---|
| 1 | Malaysia | Sarawak | Asajaya |
| 2 | Indonesia | South Kalimantan | Balangan |
| 3 | Indonesia | East Kalimantan | Balikpapan |
| 4 | Indonesia | South Kalimantan | Banjar |
| 5 | Indonesia | South Kalimantan | Banjar Baru |
| 6 | Indonesia | South Kalimantan | Banjarmasin |
| 7 | Indonesia | South Kalimantan | Barito Kuala |
| 8 | Indonesia | Central Kalimantan | Barito Selatan |
| 9 | Indonesia | Central Kalimantan | Barito Timur |
| 10 | Indonesia | Central Kalimantan | Barito Utara |
| 11 | Malaysia | Sarawak | Bau |
| 12 | Malaysia | Sabah | Beaufort |
| 13 | Malaysia | Sarawak | Belaga |
| 14 | Indonesia | Bangka Belitung | Belitung Timur |
| 15 | Malaysia | Sabah | Beluran |
| 16 | Indonesia | West Kalimantan | Bengkayang |
| 17 | Indonesia | East Kalimantan | Berau |
| 18 | Malaysia | Sarawak | Betong |
| 19 | Malaysia | Sarawak | Bintulu |
| 20 | Indonesia | East Kalimantan | Bontang |
| 21 | Brunei | --- | --- |
| 22 | Indonesia | North Kalimantan | Bulungan |
| 23 | Malaysia | Sarawak | Dalat |
| 24 | Malaysia | Sarawak | Daro |
| 25 | Indonesia | Central Kalimantan | Gunung Mas |
| 26 | Indonesia | South Kalimantan | Hulu Sungai Selatan |
| 27 | Indonesia | South Kalimantan | Hulu Sungai Tengah |
| 28 | Indonesia | South Kalimantan | Hulu Sungai Utara |
| 29 | Malaysia | Sarawak | Julau |
| 30 | Malaysia | Sarawak | Kanowit |
| 31 | Malaysia | Sarawak | Kapit |
| 32 | Indonesia | Central Kalimantan | Kapuas |
| 33 | Indonesia | West Kalimantan | Kapuas Hulu |
| 34 | Indonesia | Central Kalimantan | Katingan |
| 35 | Indonesia | West Kalimantan | Kayong Utara |
| 36 | Malaysia | Sabah | Keningau |
| 37 | Indonesia | West Kalimantan | Ketapang |
| 38 | Malaysia | Sabah | Kinabatangan |

| | | | |
|---|---|---|---|
| 39 | Indonesia | South Kalimantan | Kota Baru |
| 40 | Malaysia | Sabah | Kota Belud |
| 41 | Malaysia | Sabah | Kota Kinabalu |
| 42 | Malaysia | Sabah | Kota Marudu |
| 43 | Indonesia | West Kalimantan | Kota Pontianak |
| 44 | Indonesia | Central Kalimantan | Kotawaringin Barat |
| 45 | Indonesia | Central Kalimantan | Kotawaringin Timur |
| 46 | Malaysia | Sabah | Kuala Penyu |
| 47 | Indonesia | West Kalimantan | Kubu Raya |
| 48 | Malaysia | Sarawak | Kuching |
| 49 | Malaysia | Sabah | Kudat |
| 50 | Malaysia | Sabah | Kunak |
| 51 | Indonesia | East Kalimantan | Kutai Barat |
| 52 | Indonesia | East Kalimantan | Kutai Kartanegara |
| 53 | Indonesia | East Kalimantan | Kutai Timur |
| 54 | Malaysia | Labuan | Labuan |
| 55 | Malaysia | Sabah | Lahad Datu |
| 56 | Indonesia | Central Kalimantan | Lamandau |
| 57 | Indonesia | West Kalimantan | Landak |
| 58 | Malaysia | Sarawak | Lawas |
| 59 | Malaysia | Sarawak | Limbang |
| 60 | Malaysia | Sarawak | Lubok Antu |
| 61 | Malaysia | Sarawak | Lundu |
| 62 | Indonesia | North Kalimantan | Malinau |
| 63 | Malaysia | Sarawak | Marudi |
| 64 | Malaysia | Sarawak | Matu |
| 65 | Indonesia | West Kalimantan | Melawi |
| 66 | Malaysia | Sarawak | Meradong |
| 67 | Malaysia | Sarawak | Miri |
| 68 | Malaysia | Sarawak | Mukah |
| 69 | Indonesia | Central Kalimantan | Murung Raya |
| 70 | Malaysia | Sabah | Nabawan |
| 71 | Indonesia | Kepulauan Riau | Natuna |
| 72 | Indonesia | North Kalimantan | Nunukan |
| 73 | Malaysia | Sarawak | Pakan |
| 74 | Indonesia | Central Kalimantan | Palangka Raya |
| 75 | Malaysia | Sabah | Papar |
| 76 | Indonesia | East Kalimantan | Paser |
| 77 | Indonesia | East Kalimantan | Penajam Paser Utara |
| 78 | Malaysia | Sabah | Penampang |
| 79 | Malaysia | Sabah | Pitas |

| | | | |
|---|---|---|---|
| 80 | Indonesia | West Kalimantan | Pontianak |
| 81 | Indonesia | Central Kalimantan | Pulang Pisau |
| 82 | Malaysia | Sabah | Putatan |
| 83 | Malaysia | Sabah | Ranau |
| 84 | Malaysia | Sarawak | Samarahan |
| 85 | Indonesia | East Kalimantan | Samarinda |
| 86 | Indonesia | West Kalimantan | Sambas |
| 87 | Malaysia | Sabah | Sandakan |
| 88 | Indonesia | West Kalimantan | Sanggau |
| 89 | Malaysia | Sarawak | Saratok |
| 90 | Malaysia | Sarawak | Sarikei |
| 91 | Indonesia | West Kalimantan | Sekadau |
| 92 | Malaysia | Sarawak | Selangau |
| 93 | Malaysia | Sabah | Semporna |
| 94 | Malaysia | Sarawak | Serian |
| 95 | Indonesia | Central Kalimantan | Seruyan |
| 96 | Malaysia | Sarawak | Sibu |
| 97 | Malaysia | Sarawak | Simunjan |
| 98 | Indonesia | West Kalimantan | Singkawang |
| 99 | Indonesia | West Kalimantan | Sintang |
| 100 | Malaysia | Sabah | Sipitang |
| 101 | Malaysia | Sarawak | Song |
| 102 | Malaysia | Sarawak | Sri Aman |
| 103 | Indonesia | Central Kalimantan | Sukamara |
| 104 | Indonesia | South Kalimantan | Tabalong |
| 105 | Malaysia | Sabah | Tambunan |
| 106 | Indonesia | North Kalimantan | Tana Tidung |
| 107 | Indonesia | South Kalimantan | Tanah Bumbu |
| 108 | Indonesia | South Kalimantan | Tanah Laut |
| 109 | Indonesia | South Kalimantan | Tapin |
| 110 | Indonesia | North Kalimantan | Tarakan |
| 111 | Malaysia | Sarawak | Tatau |
| 112 | Malaysia | Sabah | Tawau |
| 113 | Malaysia | Sabah | Tenom |
| 114 | Malaysia | Sabah | Tongod |
| 115 | Malaysia | Sabah | Tuaran |
